# Design of Arbitrary Magnetic Patterns on Magnetic Polymer Composite Objects: A Finite Element Modelling Tool

**DOI:** 10.3390/polym14183713

**Published:** 2022-09-06

**Authors:** Pedro González-Losada, Marco Martins, Elvira Paz, K. B. Vinayakumar, Domingos Pereira, Ana R. Cortez, Diogo Elói Aguiam

**Affiliations:** 1 International Iberian Nanotechnology Laboratory, 4700-300 Braga, Portugal; 2 CELOPLÁS, Plásticos para a Indústria S.A., Rua de S. Mateus, 4775-127 Grimancelos, Portugal

**Keywords:** finite element method, magnetic polymer composite, magnetic sensor, magnetization

## Abstract

Magnetic sensor systems integrate a sensing element and magnetic field generators to determine their relative position or to measure movement. Typically, the magnetic fields are produced by permanent magnets, which have high intensity but are hard to machine into custom shapes. However, novel solutions using magnetic polymer composites (MPCs) have emerged as field generators due to their low cost, weight and patterning freedom. Here, we present a finite element model developed in COMSOL Multiphysics that allows the design of complex magnetization patterns on these polymer composites, taking into account the geometries of the parts and the magnetic properties of the materials employed. The model, together with the characterization protocol of the materials, has proved to be capable of predicting the magnetization of polymer composites at different temperatures. In addition, the model incorporates the properties of the magnets used during the magnetization process, such as the size, shape and magnetization, as well as the properties of the surrounding elements. This new model facilitates the design of new polymeric parts with complex shapes and magnetization patterns that can be employed as field generators in magnetic sensing systems.

## 1. Introduction

The use of magnetic sensors has increased exponentially because new industrial applications, such as automotive, robotics or biomedical, demand more and more sensing capabilities [1,2,3,4]. In the particular case of the automotive industry, among all the sensors present in a vehicle, magnetic sensors are ideal for contactless position, angle or rotational speed sensing due to their high performance, robustness in harsh environments and low fabrication cost [5,6,7]. The magnetic fields detected by these sensors are produced by strong permanent magnets, typically containing rare earth materials, attached to the moving mechanism. The geometries of these permanent magnets are limited by the trade-off between the manufacturing complexity (sintering and casting, machining and part magnetization), cost and the required magnetic performance, restricting most applications to using simple designs of high-density magnets, such as rods or blocks [8]. As an alternative to permanent magnets, a more sustainable and flexible option consists of magnetic polymer composites (MPCs), which are used to produce functional magnetized parts with complex geometries, such as gears, rings, encoder wheels or shafts, that generate the magnetic field without the need of a supplementary permanent magnet in these sensor systems [9,10,11,12].

The MPCs used to produce these polymer-bonded magnets consist of a magnetic filler embedded in a thermoplastic matrix. The matrix employed is usually a polymer with a high melt flow rate—such as nylons—to make them suitable for injection moulding. Concerning the magnetic filler, several types are used, including isotropic and anisotropic micron-sized particles of strontium ferrite (SrFe_12_O_19_), barium ferrite (BaFe_12_O_19_), samarium cobalt (SmCo) or neodymium iron boron (NdFeB) [11,13,14]. Although the 3D-printing of MPCs has recently been reported [15,16,17,18], they are mainly processed using injection moulding due to its cost-effective character and the very narrow tolerances achieved [19,20].

In comparison to conventionally produced sintered magnets, polymer-bonded magnets are lighter and less brittle, and the injection moulding processes enable the production of parts in high quantities. Additionally, their production offers a greater freedom of design regarding shape and magnetizing structure. However, they present lower magnetic performances, because their density of magnetic material is lower (70 to 90%) compared to traditional sintered permanent magnets (>95%) [8].

**Figure 1:**
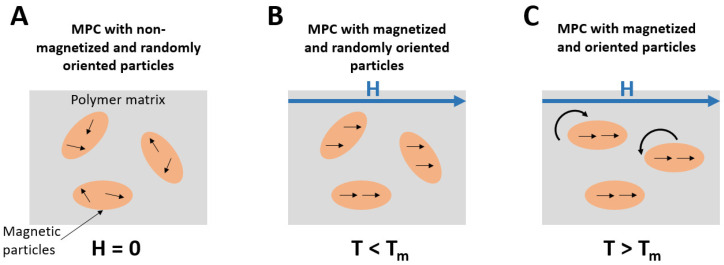
Orientation of the magnetic particles in the MPC under different conditions: (**A**) randomly oriented particles embedded in the polymeric matrix without any applied field (H = 0) and temperature under the melting temperature of the polymer (T_m_); (**B**) when an magnetizing field strong enough (H > H_s_) is applied and the temperature is under the melting temperature of the polymer, the particles are magnetized but not oriented; (**C**) when a strong enough magnetizing field (H > H_s_) is applied and the temperature is over the melting temperature of the polymer, the particles are magnetized and oriented in the direction of the magnetizing field.

In the virgin configuration, the magnetic moments of the magnetic particles are randomly oriented (see Figure 1A), and the overall magnetization is null (B = 0). When an external magnetic field (H) is applied, the magnetic moments align parallel to it (see Figure 1B) and, if the field is high enough (H > Hs), all the moments become parallel to the field and the saturation state is reached (B_s_). After removing the magnetizing field (H = 0), part of the magnetic moments stay aligned, and the value of the magnetization decreases to a remanence value (B_r_). In addition, in the case of anisotropic magnetic particles, if a magnetic field is applied above the melting point of the polymer matrix, not only the magnetic moments align along the field direction but also the particles themselves. The easy axes of all the particles align, increasing the saturation magnetization and thus decreasing the saturation field.

The final configuration of the particles—in terms of orientation and magnetization— determines the magnetic properties of the MPC. Both anisotropy and magnetic moment alignment can occur in one step, with a high field and elevated temperature during the injection of the polymer in the moulding tool. Or it can occur separately, first orienting the particles during injection, followed by a second stronger external field to align the magnetic moments. This process depends on the properties of the magnet, MPC and surrounding materials, as well as geometric factors.

When designing magnetic sensor systems, the magnetic field generated by the magnetic source (e.g., permanent magnet or MPC) must be properly determined to select the most adequate sensor in terms of sensitivity, dynamic range, number of axes or positioning distance. This is typically performed either using analytical models for simple magnet geometries, or using finite element method (FEM) models that can determine the magnetic fields in the case of complex patterns or geometries [21]. However, when using MPCs as magnetic field sources, the magnetic field they generate depends on the MPC magnetization stage, defined by the alignment process described above. The magnetization curve of the MPCs presents a non-linear behaviour that needs to be considered by the model in order to design the magnetic source adapted to the requirements of the application. Current FEM models do not provide a reliable simulation of the magnetic field generated by the MPCs considering the moulding and magnetization process.

In this work, we propose a finite element method model to design arbitrary magnetic patterns on magnetic polymer composite objects. The model, implemented in COMSOL Multiphysics, is described, as well as the characterization process of the MPC required to effectively model its behaviour. In addition, magnetization experiments under different conditions (temperature, magnet size and position) were performed and compared to the results from the FEM model to assess its validity. Finally, we present an experimental application example of a magnetic sensor system using a single MPC part with an encoded magnetization, illustrating how the FEM model is used to design and realistically simulate the field produced by the MPC part.

## 2. Finite Element Method (FEM) Model

The magnetization process of injection-moulded MPC parts is performed using a magnetic source that provides the necessary field to magnetize the particles of the composite. While magnetic field sources can be either electromagnets or permanent magnets, the latter is preferred, particularly in the case of injection moulding systems, due to concentrated field strength and reduced size.

Simple magnet geometries and magnetizations can be analytically determined. However, analytical models present some limitations when modelling the magnetization of MPCs with arbitrary patterns. First, when complex magnet configurations are required to achieve the desired magnetization pattern—e.g., arrays used for encoders—analytical models may not be available. Second, the non-linear behaviour of the materials—such as the MPC’s initial magnetization—cannot be taken into account using analytical methods. Finally, when magnetizing complex-shaped objects such as shafts, gears or encoders, analytical methods cannot describe the interaction between the object and the magnetic field.

**Figure 2:**
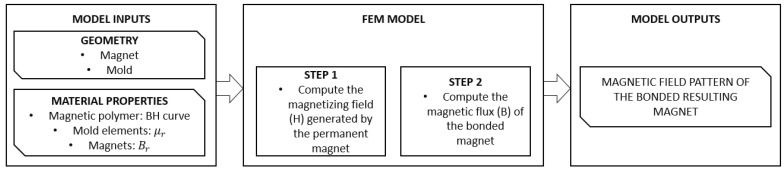
Workflow of the proposed FEM model.

In this context, the finite element method (FEM) represents a unique tool to model all these aspects in a versatile manner. FEM is a numerical method that divides the problem into smaller domains—finite elements—over which the solution is computed according to the physical equations defined. In this work, we propose an FEM model workflow (illustrated in Figure 2) to aid in the magnetization design and evaluation of magnetic performance of injection-moulded MPCs. In the case here presented, the FEM must model two different aspects of the process: on the one hand, the magnetizing field generated by the permanent magnet arrangement and used for the magnetization of the MPC part; and on the other hand, the resulting magnetic field of the bonded magnet.

The model takes into consideration: (1) the geometries of the permanent magnets used to magnetize the magnetic polymer, (2) the geometry of the bonded object and (3) the properties of the different materials of the parts involved in the process: mould, magnet and MPCs. For this, as shown in the workflow (Figure 2), the model is divided in two sequential computing steps: first, the magnetizing field (H) generated by the permanent magnet taking into consideration the surrounding mould materials and its interaction with the MPCs; secondly, the magnetic flux density pattern produced on the moulded object.

The model was developed using COMSOL Multiphysics and the “Magnetic fields, No Currents” interface of its AC–DC module.

The constitutive relations used to model the magnetizing field generated by an arbitrary magnet arrangement and its interaction with the surrounding materials are summarized in Table 1. The flux of the permanent magnets used for the magnetization is modelled using the “remanent flux density” constitutive relation, which models the magnetic flux as a function of the remanent flux density (B_r_, the flux density when no magnetic field is present) and the recoil permeability (μ_rec_), provided in the magnet technical datasheets. The surrounding mould material is considered to be linear and thus it is modelled using the “relative permeability” constitutive relation where the dependence between H and B is linear, and it is related to the relative permeability of the material (µ_r_). Previous constitutive relations model the behaviour of the linear elements. However, the magnetic polymer presents a nonlinear relation between B and H that is modelled with the “B–H curve” constitutive relation, which relates the magnetic flux density, B, and magnetizing field, H, as |B| = f(|H|). Thus, the initial magnetization curves that model the behaviour of the MPC before being magnetized are used as the input argument.

The second part of the study consist of computing the magnetic flux density generated by the polymer part. In this case, the polymer is considered to be surrounded by an isotropic environment (air, µ_r_ = 1) and it is modelled using a “remanent flux density” constitutive relation, where the remanent flux is the magnetic flux density, computed in the previous step, as a function of the applied magnetic field (Table 2).

## 3. Materials and Methods

### 3.1. Magnetic Polymer Composite (MPC)

Two magnetic polymer composites (MPC1 and MPC2) were employed in this work, composed of anisotropic strontium ferrite particles (aspect ratio 10 × 7) as filler material embedded in a binder matrix of PA12. The percentage of magnetic particles of these compounds is 82% for MPC1 and 85% for MPC2.

### 3.2. Magnetic Characterization of MPCs

The magnetic characterization was performed using a vibrating sample magnetometer (VSM–EV9 from MicroSense, Lowell, USA), equipped with an oven that can heat up to 700 °C. The remanence of the minor loops of the virgin magnetic polymer composite was measured to trace a curve of remanence as a function of the applied magnetic field for different temperatures.

For this, a sample was demagnetized using a protocol that applies an amplitude-decreasing alternating field until the magnetization is close to zero. A field was then applied and brought back to zero to measure the remanence. This procedure was repeated in steps of 40 kA/m up to a maximum field of 800 kA/m, which is high enough to saturate the magnetic particles. The remanences as function of the applied fields were then plotted for different temperatures: from 50 °C to 210 °C.

### 3.3. Thermoanalytic Characterization of MPCs

The heating and cooling response of the MPC was characterized using differential scanning calorimetry (DSC) to obtain the thermal transition temperatures of the material. Thus, the melting (T_m_) and crystallization (T_ct_) temperatures were measured according to ISO 11357-3 norm.

### 3.4. Magnetization Process

The magnetization process, used to validate the model against experimental data, is performed using different permanent magnets (neodymium N45 grade, from Supermagnete) to magnetize MPCs samples consisting of 12 mm diameter and 2 mm thick disks. These MPCs samples were heated in an infrared heater for 5 min at the desired temperature (50 °C, 75 °C, 100 °C, 125 °C, 150 °C, 180 °C) and magnetized afterwards by placing the permanent magnet close to the sample while it cooled down. Temperatures above 180 °C were not reached during the process to prevent their physical deformation.

### 3.5. Magnetization Characterization

The characterization of the magnetized polymer samples was performed using a magnetic linear Hall-effect sensor (ALS31300 3D from Allegro) mounted in a three-axes stage that performs scans either parallel to the surface of the sample or perpendicular to it. The minimum achievable incremental movement was 0.05 µm and the range of the magnetic probe was ±100 mT.

### 3.6. COMSOL Multiphysics Model

The input parameters used in the model are summarized in Table 3.

## 4. Results and Discussion

### 4.1. MPC Characterization

**Figure 3:**
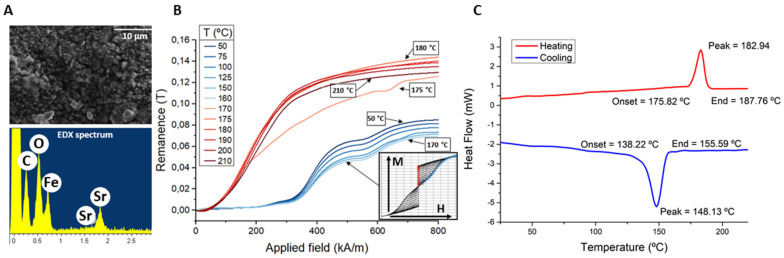
Characterization of the MPC1 used in the experiments: (**A**) SEM image of the surface of the polymer that shows the size of the magnetic particles and EDX analysis that shows the composition of the filler (SrFe); (**B**) remanence magnetization vs. applied field curve measured at different temperatures (inset: minor loops from where remanence was extracted); (**C**) DSC thermogram of the MPC.

As shown in Figure 3A, the magnetic filler of the MPCs is made of strontium ferrite particles (Sr and Fe peaks in the EDX analysis) with a size around 1–2 µm. This characterization was performed using scanning electron microscopy (SEM) to measure the particle size, as well as energy dispersive X-ray (EDX) to verify their composition.

The MPCs were magnetically characterized at different temperatures, ranging from 50 °C to 210 °C, to evaluate the temperature dependence of the magnetization. Figure 3B shows the remanence magnetization vs. field curves at the different temperatures obtained from the minor loops (inset), as described in Section 3.2. Each ordinate value corresponds to the remanence magnetization obtained for the different fields.

These curves present two well differentiated regions: one at low temperature—from 50 °C to 160 °C—and one at higher temperatures. In the low temperature region, the polymeric matrix presents a solid-state structure that prevents the embedded magnetic particles from moving in response to the applied magnetic field. This, in addition to the anisotropic characteristic of the magnetic filler, limits the total magnetization of the composite, as shown by the curves up to 170 °C of Figure 3B. Over 175 °C, the polymeric matrix reaches its melting temperature (T_m_)—see DSC thermogram in Figure 3C—allowing the magnetic particles to rotate and align with the external field, thus showing a fast transition from low to high remanence values. This increase is particularly noticeable for low values of the applied field that are not enough to fully magnetize the particles but can orient them.

We also observed a decrease of the saturation magnetization with the temperature. This is due to the decrease of the magnetization of the ferrite magnetic particles, which is around 0.2%/°C [11,22].

Thus, the magnetic characterization of the composite at different temperatures provides a method to characterize the magnetization process of any MPC. In addition, it takes into consideration the non-linear behaviour of the composite—due to the magnetic properties of the particles and their orientation—allowing more precise simulations of the magnetization at high fields.

### 4.2. Validation of the Model against Experimental Results

The data obtained from the magnetic characterization of the MPC were introduced into the FEM model (as the “B–H curve” constitutive relation) to predict the magnetization pattern of the part depending on the type of magnet employed.

In order to validate the model, we used the protocols described in Section 3.4 to magnetize a polymer disk and the protocol in Section 3.5 to characterize its magnetization pattern by scanning either parallel to the surface of the MPC disk (at 1 mm distance) or perpendicularly to it.

First, two magnets (4 mm diameter and 10 mm height), with different magnetization (axial vs. diametric), were used to magnetize the disks, which were then characterized by a surface scan at 1 mm distance.

**Figure 4:**
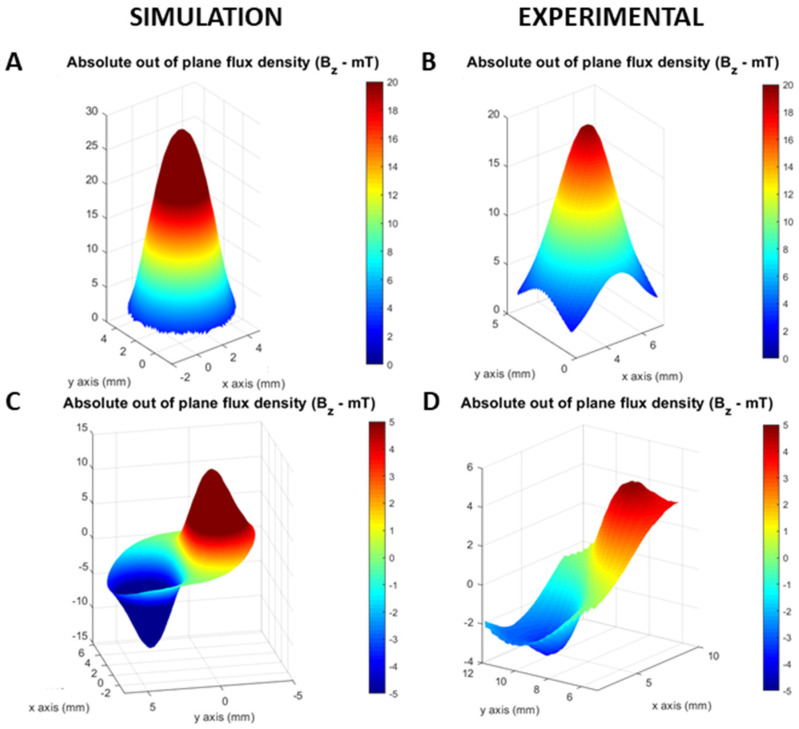
Magnetic polymer composite characterization: (**A**) measurement of the part magnetized using the axial magnet; (**B**) simulation of the part magnetized using the axial magnet; (**C**) measurement of the part magnetized using the diametric magnet; (**D**) simulation of the part magnetized using the diametric magnet.

Figure 4A,C shows the modelled absolute out-of-plane magnetic flux density (B_z_) pattern of the MPC disk obtained using an axial (Figure 4A) and diametric (Figure 4C) magnet. Figure 4B,D show the corresponding experimental values. Both simulation and measurement show a remarkably similar shape: a single pole in the axial and a double pole in the diametric configuration.

This result shows that the model can predict the type of magnetization pattern in the final piece, which constitutes an important feature, for example, in the case of position sensors.

In addition to the magnetization pattern, the absolute value of the magnetization needs to be compared to assess the validity of the model. For this, several experiments were conducted using axially magnetized magnets to magnetize the sample polymer disks under different conditions, where the maximum value of magnetic flux density produced by the magnetized part was measured on the parallel plane at 1 mm from its surface and compared with the simulated value.

**Figure 5:**
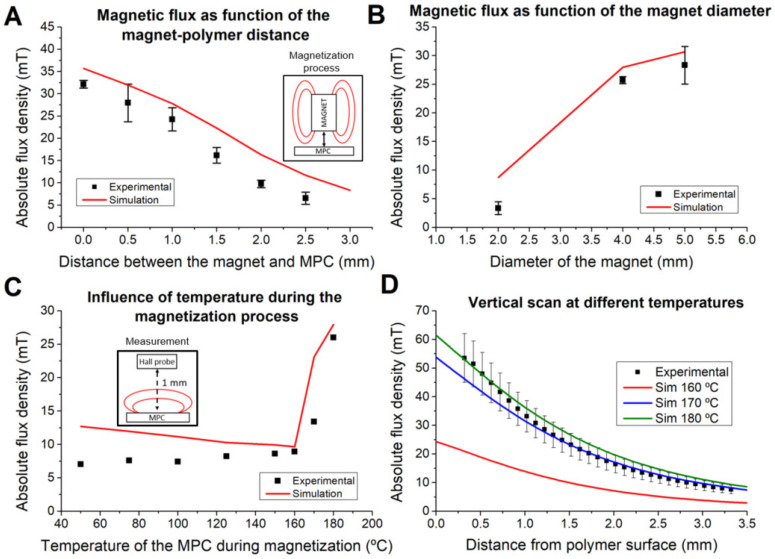
Characterization of different variables influencing the magnetization process: (**A**) influence of the distance between the magnet and the polymer during the magnetization process; (**B**) influence of the diameter of the magnet used for the magnetization; (**C**) influence of the temperature of the polymer during its magnetization; (**D**) vertical scan from the surface of the magnetic part: measured values vs. simulations at different temperatures—160 °C (**A**), 170 °C (**B**) and 180 °C (**C**).

The influence of the distance between the magnet and the polymer disk during the magnetization is shown in Figure 5A, where the resulting magnetic flux density of the disk as a function of this distance is shown. As expected, the value of the flux density decreases with the distance, showing a constant discrepancy between experimental and simulation values of 3.5 to 5.5 mT. The same effect is seen in Figure 5B, where the measured field depending on the diameter of the magnet used is plotted. For this, three different sizes were used: 2 mm, 4 mm and 5 mm. Several factors may have an influence in this discrepancy, such as a lower magnetizing field due to the temperature of the magnet. In addition, a mismatch between the distance of the MPC and the sensor can also contribute to this discrepancy.

### 4.3. Influence of the Temperature of the MPC on the Magnetization

The characterization of the material at different temperatures allowed us to simulate the magnetization process correctly for these temperatures. Figure 5C shows the absolute flux density of the MPC part magnetized at different temperatures. As expected, according to the characterization shown in Section 4.1, magnetization below 160 °C provides extremely low magnetic flux values, and it only increases when the temperature reaches 175 °C, the onset of the melting temperature of the polymeric matrix, allowing the alignment of the magnetic particles.

In Figure 5D, a vertical scan performed on a disk magnetized using a 4 mm diameter magnet with axial magnetization is compared to the simulations performed using the profile of the MPC at three different temperatures: 160 °C, 170 °C and 180 °C. As expected, the simulation curves corresponding to temperatures under the melting point of the MPC present important deviation with respect to the experimental data. Over the melting point (between 170 °C and 180 °C curves) the deviation is reduced, thus better modelling the behaviour of the MPC.

These results show that the characterization of the polymer constitutes a valuable tool to accurately predict the magnetization of the final MPC.

### 4.4. Influence of the MPC Compound and the Sourrounding Material

Besides assessing the influence of the temperature of the MPC during its magnetization, one of the objectives of this work is to provide a tool and a protocol to effectively model different magnetization patterns on distinct types of composites and different surrounding materials.

**Figure 6:**
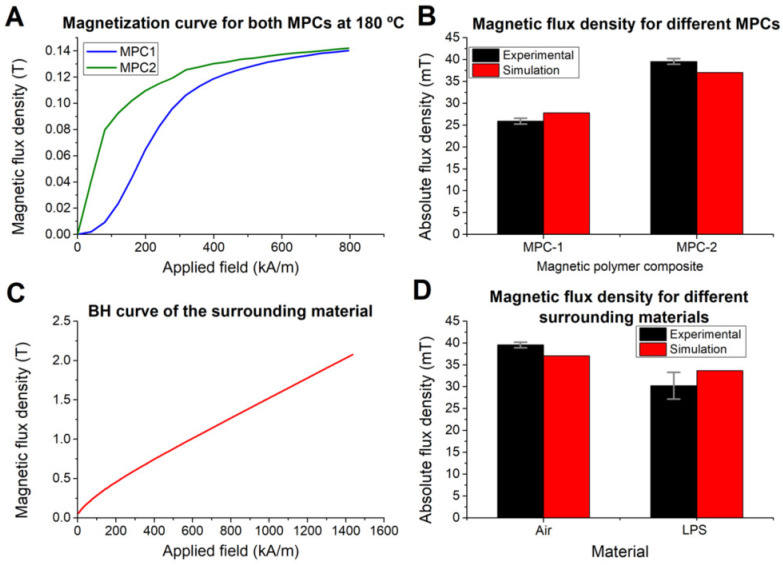
(**A**,**B**). Validation of the model for different MPCs: (**A**) initial magnetization curves for MPC-1 and MPC-2; (**B**) experimental (black bar) and simulation (red bar) magnetic flux density of the parts made of MPC-1 and MPC-2. (**C**,**D**). Validation of the model to predict the influence of surrounding materials: (**C**) first quadrant of the B–H curve of the low permeability steel disk used as spacer; (**D**) experimental (black bar) and simulation (red bar) magnetic flux density of the parts magnetized using an air gap or a metal disk.

Thus, we characterized another polymer composite (MPC2) with a higher content of strontium ferrite particles as filler (85%). As we can see in Figure 6A, the initial magnetization curve of the new compound is higher than the MPC1 due to this higher content of magnetic filler [23]. Therefore, the values of magnetic flux density in the magnetized parts are higher for the new composite, as shown by the experimental and the simulation results in Figure 6B, which represent the maximum values of magnetic flux density measured at 1 mm from the surface for both compounds after being magnetized with a 4 mm diameter magnet.

Several metals are employed in the fabrication of the injection moulding equipment that can interact with the magnetic fields and affect the final magnetization values. Hence, it is important to determine the ability of the model to effectively predict this behaviour. In the precedent experiments, the medium between the MPC disk and the magnets during the magnetization was air, which acts as a linear medium and is modelled using a constant relative permeability (µ_r_ = 1). When the surrounding materials present ferromagnetic properties, these need to be modelled accordingly. In this case, we used the B–H constitutive relation, which associates each value of the applied magnetic field (H) to a different value of the magnetic flux (B). Thus, the metallic part employed—a 6 mm diameter 1 mm thickness disk of low permeability steel (LPS)—was characterized in the VSM, and the first quadrant of the B–H curve (Figure 6C) was introduced in the COMSOL model.

For the experimental magnetization, a low permeability steel disk was used as a spacer between the magnet and the MPC. Simulation results show a reduction in the field obtained using the LPS spacer that matches the behaviour of the experimental data (Figure 6D).

These results confirm the versatility of the model, together with the characterization protocol to effectively model the magnetization process of any magnetic polymer composite or surrounding material.

### 4.5. Discussion and Model Limitations

As previously stated, this model represents a useful tool in the design of injection-moulded magnetic parts. The magnetic properties of polymer-bonded permanent magnets produced with this technology are determined by several parameters, such as the characteristics of the compound (filler content and properties, matrix material, rheological properties of the composite); the characteristics of the tool (magnetic properties of the materials, geometry, thermal properties) and the characteristics of the process (pressure, temperature, injection points) [9,11,24].

Jung et al. [25] developed an analytical model to predict the orientation of the magnetic particles in an injection moulding process coupling the hydrodynamic force, external magnetic force and internal dipole–dipole magnetic interaction. Our approach does not model the microscopic behaviour of the magnetic filler, but it characterizes the magnetic properties of the composite and uses them to predict the magnetization of the final part. 

In a simulation work, Schliesch et al. [26] developed a model to predict the holding torque generated by an eight-pole polymer-bonded rotor. In their study, they also used the empirical data from the material to feed the model, but they did not study the influence of the temperature of the material.

As in any modelling tool, the one presented in this work has several limitations that need to be taken into consideration. In particular, it cannot model several aspects of the injection moulding process that have been shown to have an influence in the final magnetization of the part, such as the flow of the polymer in the injection cavity [27], the location and number of injection points [10] or the cooling speed of the tool [28]. This is a consequence of the macroscopic character of the model, which does not predict the individual orientation of the magnetic particles, but it does consider the resulting total magnetization due to the orientation and magnetization of the particles.

In addition, small divergences between the model and the simulations are mainly due to the differences between the experimental set points and the inputs in the model of the temperature and position.

## 5. Application

After assessing the performance and limitations of the model, we explored an application where the prediction of the magnetization of the MPC is relevant. For this, we defined a magnetization pattern that can be used as an encoder to determine the angular position or velocity of the part. This pattern consists of four cylindrical magnets (2 mm diameter, Br = 1.35 mT), arranged concentrically in an antisymmetric magnetization configuration.

**Figure 7:**
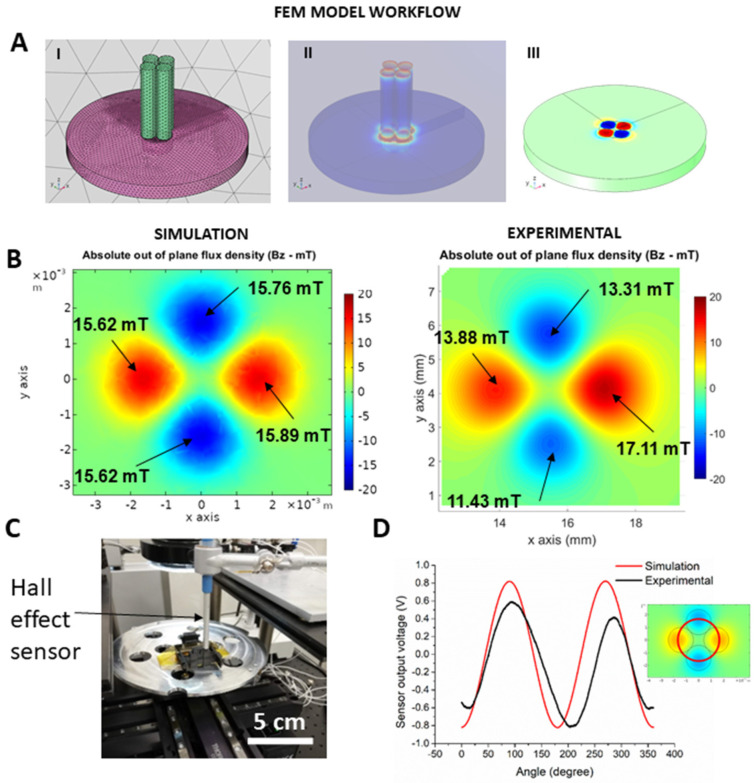
Use of the model for a magnetic encoder application: (**A**) Simulation of the magnetization pattern: (I) object drawing, material assignment and mesh generation; (II) computation of the magnetic field generated by the magnet arrangement; (III) computation of the magnetic field generated by the magnetized MPC part. (**B**). Comparison of the simulated flux density and experimental measurements at 1 mm from the surface of the MPC object. (**C**). Measurement setup with rotation stage and Hall sensor. (**D**). Measured values at the same distance from the surface.

Using the FEM model, the magnet arrangement was designed and meshed (Figure 7A(I)), then the magnetizing field of the arrangement was computed (Figure 7A(II)) and the magnetization of the MPC calculated (Figure 7A(III)). The results of the simulation (Figure 7B left) were compared to the experimental results obtained from the characterization of the MPC at 1 mm from its surface (Figure 7B right). Some differences appear in the magnetization values, mainly due to the manual positioning of the magnets during the magnetization process and the tolerances in the magnetization of the magnets.

A Hall-effect sensor with a sensitivity of 100 mV/mT and a range of 35 mT—compatible with the values obtained in the simulations—was placed at 1.5 mm from the surface of the MPC part to emulate an angular position sensor system. The magnetized part was then placed in a rotation stage, and the signal of the sensor was continuously rotated and recorded using a gaussmeter (Lakeshore DSP 475) connected through an analogue-to-digital converter to the computer. The resulting signal (Figure 7D black line) was in the same range of the simulations output signal (Figure 7D red line) along the defined trajectory (Figure 7D, inset). The main differences are due to the small deviation of the magnetization process already observed in Figure 7B and the error in the eccentric positioning of the sensor.

The values of the simulations demonstrate their validity to predict a complex magnetization pattern. In addition, the model can assist in the design of the whole sensing system by providing an estimation of the expected signal, which is used to set the requirements for the electronic readout system.

## 6. Conclusions

In this work, we presented a finite element model as well as a material characterization protocol that allows one to predict the magnetization characteristics of any of magnetic polymer composite. The model was shown to precisely predict the shape of the magnetization pattern as well as the absolute value of the magnetic flux density of the magnetized MPC. This was evaluated using different magnet configurations (size, magnetization and distance from polymer), showing a good match between the simulation and experimental results.

In addition, the effect of temperature was also studied thanks to the magnetic characterization of the polymer at different temperatures, making this tool useful in injection moulding applications or additive manufacturing.

The model was also validated for two different materials with different filler content and proved to effectively estimate the final magnetization.

Finally, the model was used to design a simple magnetic encoder using a complex magnetization pattern consisting of an array of four cylindrical magnets arranged in an antisymmetric configuration, showing that it allowed us to predict the output voltage of the sensor.

This work opens the door to the fast and precise design of arbitrary magnetic sensing systems where the magnetic field source can be designed by selecting the material, geometry and magnetization pattern suitable for the required application.

## Figures and Tables

**Table 1:** Constitutive relations used in the first part of the model.

Element	Constitutive Relation	Equation
Magnets	Remanent flux density	$B=\mu_{0}\mu_{r}H+B_{r}$
Surrounding materials	Relative permeability	$B=\mu_{0}\mu_{r}H$
Magnetic polymer	B–H curve	$B=f\left(\left|H\right|\right)\frac{H}{\left|H\right|}$

**Table 2:** Constitutive relations used in the second part of the model.

Element	Constitutive Relation	Equation
Magnetic polymer	Remanent flux density	$B=\mu_{0}\mu_{\mathrm{rec}}H+B_{r}$
Environment	Relative permeability	$B=\mu_{0}\mu_{r}H$

**Table 3:** Properties of the materials used in the COMSOL^®^ model.

Part	Property	Value
Permanent magnet	Br	1.35 T
Surrounding environment (air)	µ_r_	1
Vacuum permeability	µ_0_	4π × 10^−5^ H/m
Recoil permeability for N45 grade magnets	µ_rec_	1.04

## Data Availability

The data that support the findings of this study are available from the authors, upon reasonable request.
